# 不忘初心  砥砺前行

**DOI:** 10.3779/j.issn.1009-3419.2018.03.01

**Published:** 2018-03-20

**Authors:** 捷 赫

**Affiliations:** 100021 北京，国家癌症中心/中国医学科学院肿瘤医院胸外科 Department of Toracic Surgery, National Cancer Center/Cancer Hospital, Chinese Academy of Medical Science, Beijing 100021, China

**Keywords:** 胸外科, 外科医师, 胸外科医师协会, Toracic surgery, Toracic surgeon, Chinese Association of Toracic Surgeon

纵观医学发展，无论国际还是国内，胸外科一直都是担负生命守护重任、令人羡慕且充满挑战的高精尖学科。时代在发展，社会在进步，对胸外科医师提出的要求也越来越高，除了具备将基础理论和临床实践融会贯通的能力，以及敏锐的观察力和坚定的意志力，还需要在学习中不断吸收新知识、吐故纳新、勇于创新。因此，一名胸外科医师从筛选培养、到发展成熟，绝对不是一蹴而就的简单过程。作为胸外科医师的一员，作为生命健康的坚定捍卫者和守护者，我们在感到自豪的同时，更应深知责任重大、使命光荣。

胸外科医师协会既是胸外科医师学习交流、互通有无的平台，也是想其所盼、得其所求的暖心的"家"。新时代要跑出新的加速度，胸外科医师协会将一如既往地发挥聚"人"、聚"气"、聚"事"的良好平台作用，团结组织广大胸外科医师，弘扬救死扶伤的人道主义职业精神，提高医师队伍建设水平，在毕业后继续教育、专科培训和定期考核等方面发挥积极作用；做好医师维权工作，致力于建设和保障医师发展环境，让广大同道学有所成、劳有所获；根据国家中长期发展规划，明确发展方向，促进胸外科事业不断向前发展。

## 一 聚"人"力、育青苗，保障我们的事业后继有人

发展是第一要务，人才则是第一资源。优秀的人才是成就事业的首要元素与保障。目前我国胸外科聚集了一大批年富力强的优秀专家，他们活跃在国际舞台之上，在胸外科的各个领域取得了瞩目的成就，受到国际同行们的高度重视与赞誉。但随着社会发展中诸多因素的出现和变化，胸外科过去那样"炙手可热"的情况有所改变。在美国，胸外科已经出现了不同程度的住院医生招收"荒"，我国的形势也不容乐观。胸外科医师协会要抢占人才战略制高点，为行业未来聚集精英，宣传胸外科专业的重要性、先进性和挑战性，号召优秀的人才致力于胸外科事业，发现和吸引优秀的医学生选择胸外科，培养、教育胸外科住院医生、主治医生成为有能力、有担当、有抱负的高端胸外科人才，从而为胸外科事业的未来发展聚拢优秀人才资源。

## 二 聚"气"氛、扬正气，保障我们的同道受人尊重

正能量的行业风气，是保证我们行业赢得社会各界尊重的前提。我国广大的胸外科医师队伍历来都是一支充满政治自信、文化自信、制度自信、专业自信的正能量的优秀队伍。但是我们也必须清醒的认识到，堤溃蚁孔、气泄针芒，社会上某些浮躁气氛对我们行业的不断侵蚀与影响。胸外科医师协会不但要团结自律，坚决遵守国家相关法律法规，更要在廉洁行医、严谨务实、求真创新等方面，引导胸外科医师做到慎独慎微，心不动于微利之诱、目不炫于五色之惑，培养功成不必在我的胸襟，为行业发展提供充满正能量的良好学术风气。

## 三 聚"事"业、重创新，保障我们的学科永立潮头

当今世界已经进入了一个新的时代，信息爆炸、人工智能、世界大联网、精准医学等成为发展风向标，已经完全有别于过去单打独斗，条块分割、信息阻遏的时代。医学证据级别也步入了多中心联合研究的大数据、高级别循证医学时代。未来胸外科的规划中心应该是"合作、精准、发展"，要跟随时代、科学发展以及人性化发展。因此，胸外科医师协会有必要在数据库建设、质量控制、行业指导、规范制定，尤其是多中心联合研究、数据资源共享等方面发挥更重要的引领作用，发挥我国病例资源多的巨大优势，积极开展多中心、大样本的临床研究。

站在新的历史起点再出发。在习近平新时代社会主义思想的指引下，胸外科医师协会将本着"服务、协调、自律、维权、监督、管理"的协会精神，牢记为人民生命健康负责的光荣使命，不忘初心，砥砺前行，不断优化资源配置、挖掘社会潜力，营造出百舸争流、千帆竞发的生动局面，把我国胸外科事业做好、做强，为我国人民的生命健康继续努力奋斗！

